# Supplementary material for: Alpine Musk Deer (Moschus chrysogaster) Adjusts to a Human-Dominated Semi-Arid Mountain Ecosystem
Source: Animals (Basel). 2022 Nov 7;12(21):3061. doi: 10.3390/ani12213061 (PMC9658949; doi:10.3390/ani12213061)
Supplement: Supplementary file 1 [file animals-12-03061-s001.zip › supplementary material.pdf]

## Supplementary Materials

**Table S1.** The percent contribution and permutation importance of environmental variables to the MaxEnt model.

| | Green grass period | | Withered grass period | |
|---|---|---|---|---|
| Environmental Variable | Percent Contribution (%) | Permutation importance | Percent Contribution (%) | Permutation importance |
| **Distance to cultivated land** | **35.6** | **20.1** | **44.0** | **45.1** |
| **Aspect** | **15.4** | 12.8 | **17.2** | **12.1** |
| **Elevation** | 6.9 | 8.0 | **16.4** | **22.2** |
| **Land coverage type** | **17.3** | **13.8** | **6.0** | 1.0 |
| **Distance to residents** | **11.1** | **18.4** | 3.4 | **9.6** |
| NDVI | 6.4 | **13.0** | 5.3 | 1.3 |
| Distance to roads | 4.2 | 9.8 | 4.4 | 7.5 |
| Slope | 3.0 | 4.1 | 3.3 | 1.3 |

Note： The first five variables with the highest percent contribution and permutation importance are presented in bold.

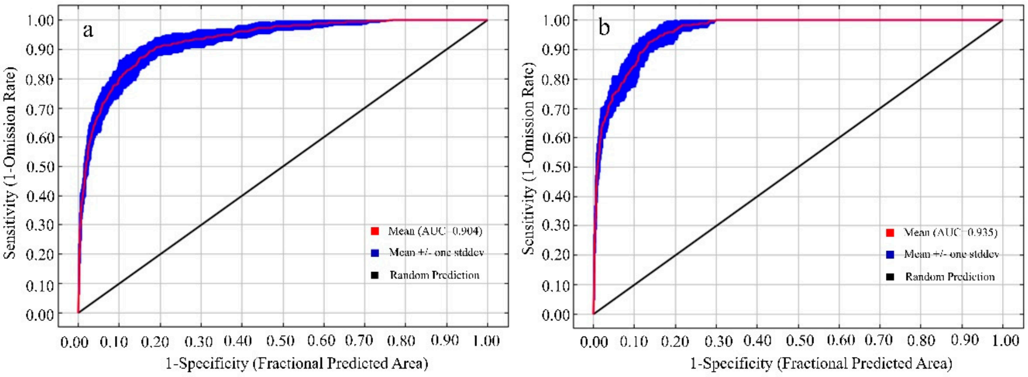


**Figure S1.** Model performance based on the area under the curve (AUC) for Alpine musk deer during (a) Green grass period and (b)withered grass period.

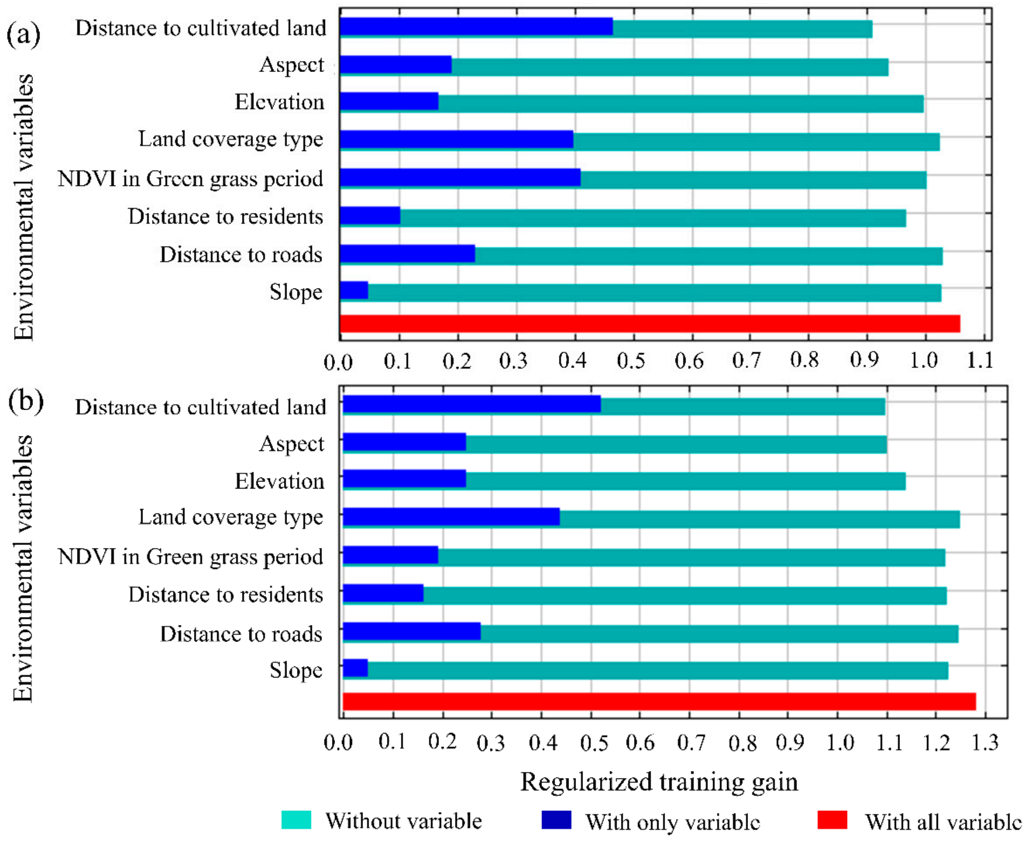


**Figure S2.** Jackknife test of the importance ranking of environmental variables by the habitat suitability models for Alpine musk deer during (a) Green grass period and (b)withered grass period. The longer the blue bar, the more important the predictor variable.

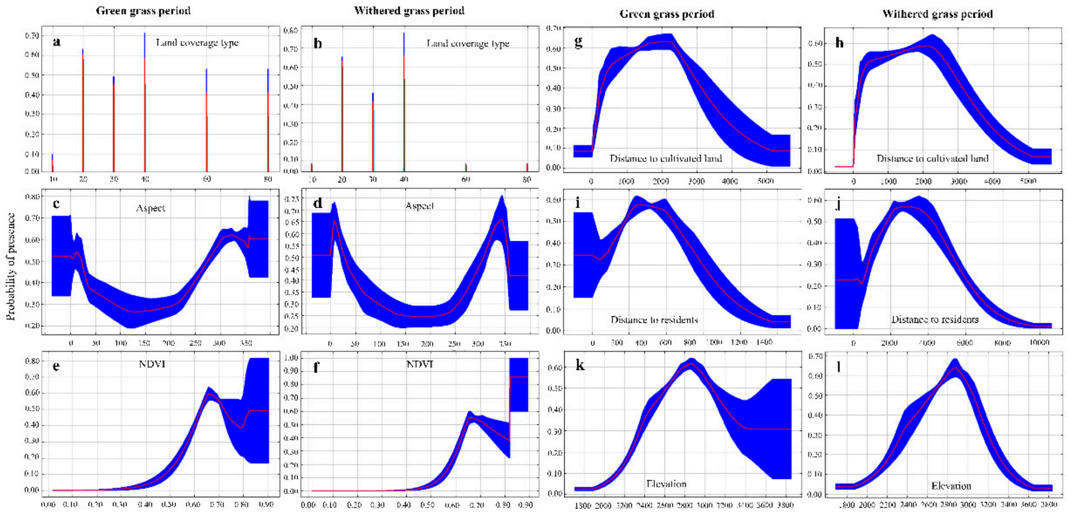


**Figure S3.** Response curve results of MaxEnt modeling of the first six environmental variables for potentially suitable habitat for Alpine musk deer including land coverage types (The abscissa 10 of the response curve of land coverage type represents cultivated land, 20 presents forest, 30 presents grassland, 40 presents shrub, 60 presents water body, and 80 presents land transformed by human activities, available online: http:// www. Globalland cover.com/) (a) during the green grass period and (b) withered grass period; Aspect (c) during the green grass period and (d) withered grass period; NDVI

(e) during the green grass period and (f) withered grass period； distance to cultivated land (g) during the green grass period and (h) withered grass period; elevation (i) during the green grass period and (j) withered grass period.
